# Persistent Median Artery, Bifid Median Nerve, and Reversed Palmaris Longus Encountered During Cadaveric Dissection: The First Reported Case

**DOI:** 10.7759/cureus.40324

**Published:** 2023-06-12

**Authors:** Asem H Elhossiny, Mohamad Bakir, Ahmad Dawalibi, Ayman Behiery

**Affiliations:** 1 College of Medicine, Alfaisal University, Riyadh, SAU; 2 Department of Anatomy, Alfaisal University, Riyadh, SAU

**Keywords:** carpel tunnel syndrome, case report, rare anatomical variants, pma, reversed palmaris longus, bifid median nerve, deep palmar arch, superficial palmar arch, persistent median artery

## Abstract

The median artery is a transient embryological structure that normally disappears with the development of the radial and ulnar arteries. In rare instances, though, it persists as the persistent median artery (PMA). The superficial and deep palmar arches are formed through the anastomoses of the radial and ulnar arteries, giving hand and digits their main blood supply. This complex network of vessels and their anastomoses are prone to anatomical variations based on how the anastomosis occurs and which arteries contribute to this anastomosis. While it normally forms through the anastomosis of the radial and ulnar arteries, the superficial palmar arch (SPA) may also form differently, as in our case here, where the median artery persisted and branched off the radial artery, anastomosing with the ulnar artery to give rise to the SPA. This may also interfere with the normal compartmental architecture within the hand, possibly contributing to various clinical pathologies like carpal tunnel syndrome (CTS). Notably, in addition to the persistent median artery, our findings revealed a reversed palmaris longus and a bifid median nerve. These two additional variations can potentially exacerbate the risk of CTS. Alone, the coexistence of the PMA and the reversed palmaris longus is deemed a rare anomaly, only reported once in the literature. The addition of a third variation to the existing ones, like the bifid median nerve, is first reported by us and calls for more investigation for a possible genetic mutation. In this case, we report a persistent median artery, reversed palmaris longus muscle, and bifid median nerve in the forearm of a male cadaver found during a routine anatomy teaching session.

## Introduction

The hand is supplied mainly through the anastomoses of the brachial artery's main branches, the radial and ulnar arteries. Both arteries carry blood across the wrist and then divide into superficial and deep branches. Those branches then anastomose to form two arches: the superficial volar arch and the deep volar arch. The superficial arch is formed from the anastomosis of the ulnar artery with the superficial branch of the radial artery, but mostly from the ulnar artery. The deep arch is formed from the anastomosis of the dorsal radial artery with the deep branch of the ulnar artery, but mainly from the dorsal radial artery [1].

The superficial arch then gives rise to three branches that supply the digits, called the common palmar digital arteries. Those branches travel between the finger webs and supply most of the digits, except for the medial aspect of the fifth digit, which is supplied by another branch of the superficial arch [2,3]. The deep arch also gives rise to three branches called the palmar metacarpal arteries. Those branches anastomose with the superficial arch’s common palmar digital arteries. Together, those two arches and their branches form a complex network of collateral supply to the hand and its structures [4,5].

This complex system of arterial anastomoses and collaterals creates room for anatomical variations. The arches can form different patterns or variations based on whether or not the anastomosis occurs. If anastomosis occurs, the arch is labeled a complete arch, and if absent, it is labeled an incomplete arch, with the complete arch accounting for the majority of cases. Seven different types of complete and five different types of incomplete superficial arches have been mentioned in the literature. Those categories and variations in the vasculature are especially important for hand surgery [6].

Embryologically, the normal pattern of the hand vasculature is believed to have developed by a combination of structures through different stages; first, the dorsal aorta gives rise to a network of capillaries that grow into limb buds at stage 12. The network’s proximal parts grow, the arterial wall differentiates, and this differentiation eventually yields the ulnar, interosseous, and median arteries. Finally, the pattern of the vessels and the arches develops. Other theories suggest that genetics, oxygenation, and hemodynamic forces play a role [1].

Normally, the median artery is a transient vessel that irrigates the embryo’s hand during an early embryonic stage and should regress afterwards with the development of the ulnar and radial arteries. In some people, however, it persists. The persistent artery can either end partially (antebrachial type) before reaching the wrist or fully (palmar type) before reaching the palm, the latter of which we label the PMA [7]. The persistence of this vessel changes how anastomoses work between the hand vessels. When present, the PMA anastomoses with the ulnar artery to give the SPA. When the persistence is partial, however, SPA does not form and thus does not anastomose with the ulnar artery. In that case, the PMA gives the common digital arteries [7,8]. In our case, though, the variant is even more rare; while the PMA is present, it is branching off the radial artery and contributing, along with the ulnar artery, to the formation of the SPA and the hand vasculature system. 

In this paper, we present a case of multiple rare variants all present in one male cadaver: a left-sided PMA branching off the radial artery, a bifid median nerve, and a reversed palmaris longus.

## Case presentation

**Figure 1:**
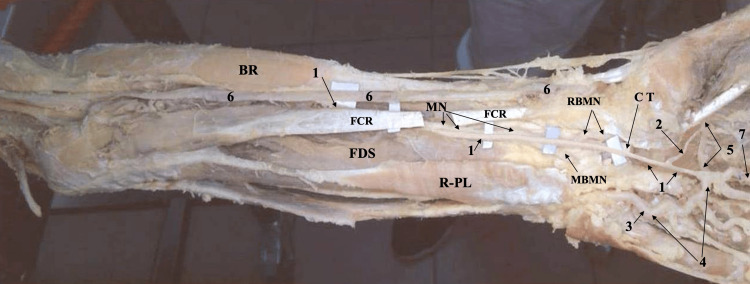
Gross image of the left forearm and hand showing the persistent median artery, bifid median nerve, and reversed palmaris longus 1. PMA, 2. A branch connecting the PMA to the DPA, 3. Ulnar artery, 4. Superficial palmar arch, 5. Deep palmar arch, 6. Radial artery, 7. The first common palmar metacarpal artery MN: Median nerve, RBMN: Radial branch of median nerve, MBMN: Medial branch of median nerve, BR: Brachioradialis muscle, FCR: Flexor carpi radialis muscle, FDS: Flexor digitorum superficialis muscle, R-PL: Reversed palmaris longus muscle, CT: Cut tendon belonging to FDS

On dissecting the SPA of a partially dissected left hand (skin and subcutaneous tissue as well as palmar aponeurosis were already removed) of a formalin-preserved cadaver, it was noticed that the SPA was complete and mainly formed by the ulnar artery, with the contribution of an artery other than the radial. Identifying the contributing vessel as the median artery-the persistent median artery-was entertained by us as it appeared to be aiming at the carpal tunnel accompanying the tendons of the flexors of the digits (superficialis and profundus) as well as the remaining parts of a mobilized median nerve. We meticulously retrieved (dissected) the contributing (median) artery proximally from its connection at the radial (lateral) end of the SPA till the proximal border of the carpal tunnel (the distal part of the forearm), sacrificing (resecting) the tendons of the digit flexors (superficialis and profundus) and the median nerve (already severed recurrent median and dissected and slightly displaced digital branches) in the area in between. This rendered the field of the palm less crowded, with structures clearly showing the SPA and the PMA running within and issuing from the carpal tunnel, descending across the palm to join the radial (lateral) end of the SPA opposite the commencement of the third common palmar digital artery (Figure 1).

**Figure 2:**
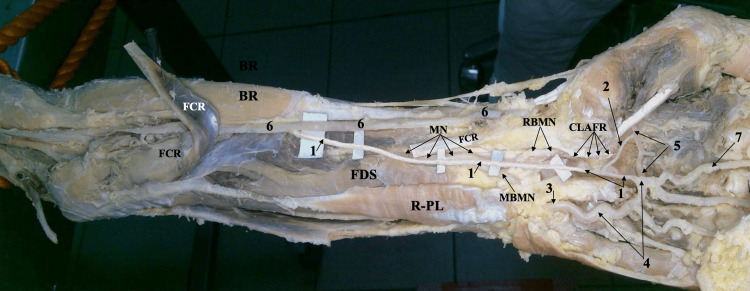
Gross image of the left forearm and hand showing the origin of the persistent median artery 1. PMA, 2. A branch connecting the PMA to the DPA, 3. Ulnar artery, 4. Superficial palmar arch, 5. Deep palmar arch, 6. Radial artery, 7. The first common palmar metacarpal artery MN: Median nerve, RBMN: Radial branch of median nerve, MBMN: Medial branch of median nerve, BR: Brachioradialis muscle, FCR: Flexor carpi radialis muscle, FDS: Flexor digitorum superficialis muscle, R-PL: Reversed palmaris longus muscle, CLARF: Cut lateral attachment of flexor retinaculum

Just after exiting the tunnel, we discovered a branch arising from the PMA heading radially (laterally) into the first web to disappear between the adductor pollicis (oblique head) and the thenar eminence (flexor pollicis brevis, the deep head). We decided to explore if there is a relationship between this branch and the deep palmar arch (DPA). For that, we proceeded with the dissection of the radial artery, exposing it from a point at the volar side of the distal forearm just before winding around to enter the snuff box until a point (at the space between the first dorsal interosseous and the adductor pollicis muscles) where it just turned into and continued as the DPA in the palm of the hand. During this dissection exercise, we removed the first dorsal interosseous and the adductor pollicis muscles; on the removal of the latter, it was obvious that the branch arising from the PMA was connecting it to the commencement of the DPA (the radial end of the arch) (Figure 2), as expected earlier.

**Figure 3:**
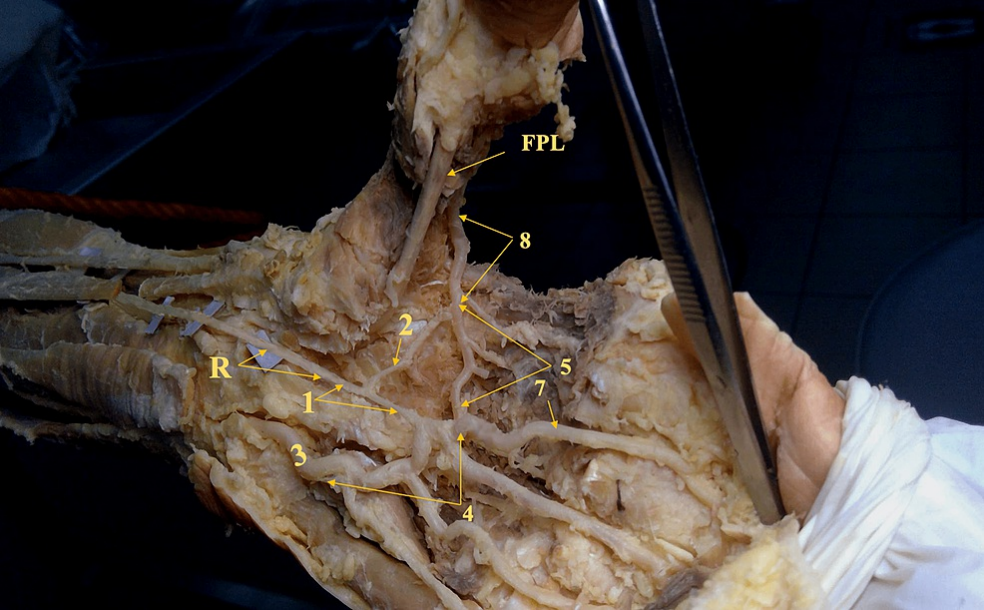
Gross image of the left hand showing the persistent median artery and its contribution to the palmar arches 1. PMA connected to the radial end of the SPA and contributing to it, 2. A branch connecting the PMA to the DPA, 3. Ulnar artery, 4. Superficial palmar arch, 5. Deep palmar arch, 6. Radial artery, 7. The first common palmar metacarpal artery, 8. Radial artery FPL: Flexor pollicis longus tendon, R: The PMA running in the carpal groove (the carpal tunnel with its flexor retinaculum removed)

Subsequently, the dissection followed progressively from the distal forearm in a proximal direction, retrieving the median artery to its origin, which was surprisingly discovered at the radial artery from a point at the mid-forearm level. During dissection, it was noticed that the median nerve was accompanying the median artery for most of its course alongside the tendons of the flexor digitorum superficialis. Also, two inches proximal to the upper end of the flexor retinaculum (removed during the procedure), it was noticed that the median nerve had split earlier into two branches. This phenomenon is known as the "bifid median nerve" and has been reported before in some other PMA cases. Certainly, the flexor carpi radialis had to be resected, and its proximal part reflected to demonstrate the full course of the PMA (Figure 3), as the artery ran deep (posterior) to the muscle after originating from the radial artery.

A video of our cadaver with a PMA originating from the radial artery, as well as a bifid median nerve and a reversed palmaris longus on the left side, is shown in Video 1.

**Video 1:** Video demonstration of the persistent median artery originating from the radial artery on the left side The reversed palmaris longus muscle and bifid median nerve can also be seen in the video.

## Discussion

The first description of the PMA was done by Sir Richard Quain in 1844; he described the artery as a small branch from the brachial artery that follows the pathway of the median nerve [9]. Over the past decades, a considerable number of papers have explored variations in the origin, course, and insertion of PMA, highlighting the relationship of this variation with medical interventions involving CTS [7]. 

Several elements may influence the final arterial structure, including unique cell adhesion molecules, mechanical stresses, transcription factors, vascular regression, and reconstruction, all of which are involved in the following phases of vasculogenesis at the beginning phases of ontogeny [7]. During the first trimester of the embryo's growth, the median and anterior interosseous arteries are the primary conduits of blood supply to the hand. The radial and ulnar arteries form after the eighth week of gestation; at that point, the median artery starts to retreat. As a result, the preservation of the median artery in an adult human could be seen as a vestige of primitive vascular design [8].

The topology of the PMA and median nerve across the forearm varies in the carpal tunnel, depending on whether it is anterior, anterolateral, or anteromedial. As a result, preoperative ultrasonography is a critical technique for avoiding iatrogenic damage during the CTS surgical procedure [10]. In most situations, the PMA anatomical variance is asymptomatic [11]. Nevertheless, it has been linked to CTS, particularly when combined with PMA aneurysm, thrombosis, or median nerve compression [12,13]. Because of the stress placed on the carpal tunnel, Salter et al. listed PMA thrombosis as one of the causes of CTS [11]. Several conditions, including an infection on the deep fascial levels, an effort done by the wrist in an atypical position, trauma, hormonal birth control pills, and prolonged wrist labor, may result in this thrombosis [11]. CTS may also result from other PMA-related causes, such as calcification, atherosclerosis, aneurysms, and trauma [14]. Surprisingly, other causes of CTS not related to PMA were also found in our case, including a bifid median nerve. Because bifid median nerves increase the mean cross-sectional area at the wrist more than non-bifid median nerves, they serve as anatomical risk factors for the emergence of CTS [15]. Additionally, PMA may be connected to other clinical conditions like pronator syndrome and anterior interosseous nerve syndrome [16]. 

Anatomical dissection was performed on 125 randomly selected isolated upper limbs. PMA was identified in five of the 125 upper limbs (4% of the total number of limbs). In three of the 125 dissected upper limbs, the PMA originated from the common interosseous artery. The PMA originated from the ulnar artery in a single case. In another instance, the PMA developed from the anterior interosseous artery [8]. Another study dissected 60 upper limbs from 30 cadavers. The PMA was detected in four limbs. In the four cases, the persistent artery originated from the anterior interosseous artery and terminated at the SPA [17]. The PMA, in our case, arose from an uncommon source, the radial artery. The persistence of the median artery emerging from the radial artery adds significant importance to our findings. It reveals a less commonly encountered pathway for this uncommon vascular variation, emphasizing the significance of our case in providing insight into the complex component of human anatomy.

The radial artery is frequently used to obtain blood gases, cannulate for arterial line placement, perform catheterizations, or harvest a graft for patients needing coronary revascularization. The blood supply to the hand stays intact if one of the arteries gets blocked during a procedure because the ulnar and radial arteries create anastomosis with the deep and superficial volar arches. Nonetheless, complications of such operations include ischemia distal to the radial artery region of puncture and limb compromise. These issues can emerge when the patient's collateral circulation fails to keep perfusion going [18].

A one-of-a-kind instance was discovered during a routine anatomical dissection in the gross anatomy department at UCLA's David Geffen School of Medicine. It featured a 72-year-old Caucasian man with bilateral persistent median arteries and a duplicated palmaris longus muscle on the right side. Both arteries originate from the ulnar artery. An extra muscle belly was discovered on the ulnar side of the typical palmaris longus in the right forearm. The aberrant muscle belly inserted on the flexor retinaculum, whereas the normal palmaris longus had a typical distal attachment. To the author's knowledge, this case is the only instance in which a right-sided double palmaris longus and a bilateral PMA have both been observed concurrently. The search terms "persistent median artery" and "palmaris longus" were used to search MEDLINE from 1920 to 2017 in order to support their claim [19]. The coexistence of reversed palmaris longus and PMA has been reported in the literature in a study published in May 2022. The authors discovered a PMA along with a reversed palmaris longus in their cadaver and claimed that this combination had never been reported in the literature before [20].

The differentiation of the muscle tissue in the forearm and hand, according to Mrázková's hypothesis, is intimately related to the establishment of the vascular bed. This implies that the blood vessels in these regions go through a process of growth and specialization at the same time the muscles do, implying that this interconnected development of vessels and muscles may demonstrate why variations in these structures may occur at the same time; any interruption or irregularity in the growth of the muscle may influence the growth of the nearby blood vessels, resulting in the development of anomalies in both structures [20]. Along with the PMA, we discovered a reversed palmaris longus in our cadaver, which was discussed extensively and published as a separate case report to ensure it was effectively elaborated on [21]. The distinctive characteristic in our case, however, was the discovery of a bifid median nerve, something that has not been previously reported in the literature. We believe that the combination of these three anomalies could be the foundation for a yet-undiscovered genetic mutation. 

In a study of 78 upper limbs collected from Australians aged 51 to 101 who died between 2015 and 2016, a total of 26 median arteries were discovered, with a prevalence rate of 33.3%. The presence of the median artery has been substantially rising (p =.001) as time passed, increasing from around 10% among individuals born in the mid-1880s to around 30% by the end of the 20th century, demonstrating microevolutionary evolution of the internal anatomy of the human body. If the current pattern keeps going, second-order polynomial regression of median artery prevalence on dates of birth indicates that individuals born 80 years from now are going to all have a median artery [22]. As a result, we believe that publishing this paper is critical, as it sheds light on a currently considered anatomical variant that could potentially evolve to be regarded as normal anatomy in the future. We believe that physicians from various specialties should be aware of this possibility to better treat patients with such anomalies and avoid complications when operating on the upper limb.

Because the hand frequently handles a wide range of chores, like grabbing items and putting digital pressure across multiple planes of movement to perform dynamic combined activities, the hand's numerous collateral circulations are critical for ensuring sufficient blood flow to all regions of the hand. Because of current evolutions in hand surgery, comprehending the vascular pattern of the hand and its variances has become critical for reliable and effective surgical results [23].

## Conclusions

The blood supply of the hand usually involves a complex system of anastomoses and collaterals through the joining of the radial and ulnar arteries and their formed arches. Any disruption in this system can have various clinical implications, like the variant in our case. Here, we discussed a persistent median artery, one of those morphological variants, and the potential impact it may have on the normal anatomy of the hand arches and its clinical significance in the literature. The persistence in our case was complete, of the median-ulnar type, anastomosing with the ulnar artery to give rise to the SPA and its terminal branches supplying the hands. Because of the close proximity the persistent artery has to the nearby structures, this morphological variation can aid in the development of certain pathologies, including pronator syndrome, interosseous nerve syndrome, and carpal tunnel syndrome. Healthcare professionals and surgeons, specifically hand surgeons, should be aware of such variations to optimize surgical planning and grafts, blood sampling, and procedures involving cannulation, all to avoid complications that could occur through those variations. The presence of a reversed palmaris longus and the bifid median nerve, along with the persistent median artery, adds confusion to the existing complexity, as this combination was not reported in the literature before, prompting further investigation of the case. 
